# Coil Orientation in Transcranial Magnetic Stimulation Affects Motor-evoked Potential Size more than its Timing or Waveform Shape

**DOI:** 10.1007/s10548-026-01233-3

**Published:** 2026-07-11

**Authors:** Dao T. A. Nguyen, Elisa Kallioniemi, Petro Julkunen, Saara M. Rissanen, Pasi A. Karjalainen

**Affiliations:** 1 Department of Technical Physics, University of Eastern Finland, Kuopio, Finland; 2 Department of Biomedical Engineering, New Jersey Institute of Technology, Newark, NJ USA; 3 Department of Clinical Neurophysiology, Kuopio University Hospital, Kuopio, Finland

**Keywords:** Transcranial Magnetic Stimulation, Motor-Evoked Potential, Coil Orientation, Feature Variation, Principal Component Analysis

## Abstract

The amplitude of the motor-evoked potentials (MEPs) induced by transcranial magnetic stimulation (TMS) is primarily influenced by the orientation of the stimulation coil, while its influence on other MEP features is not well known. Accordingly, this study investigated how the TMS coil orientation affects a comprehensive set of MEP features, including its size, timing, duration, and polyphasia. Nine healthy volunteers were recruited. Three single-pulse TMS sessions were applied to the left hemisphere’s first dorsal interosseous muscle hotspot. The coil was rotated from − 135º to 135º at this location relative to the predefined optimal stimulation angle. The first two sessions were conducted at the same hotspot, and the third at the remapped hotspot. Four levels of analysis were performed: intra-session, inter-session, inter-subject, and features grouped from all MEPs. The timing-related features of MEP were least affected by coil orientation and were intrinsic to each subject, whereas its size-related features varied significantly across subjects. The first two principal components of the MEP waveform accounted for 91.3% ± 3.0% of each dataset’s total variation, indicating intra-session consistency of MEP waveforms. MEP waveforms were comparable intra- and inter-session and slightly different across subjects. Our study provides a comprehensive analysis of how coil orientation affects MEP features waveform morphology and complements previous studies that evaluated only the effects on MEP peak-to-peak amplitude and onset latency. Size-related MEP features varied with coil orientation, which likely changes the effective stimulation intensity at the cortical target, while timing-related features and the dominant waveform morphology were less affected. Furthermore, MEP waveforms were highly consistent across sessions within subjects and differed only slightly across subjects, indicating high within-visit repeatability in healthy subjects.

## Introduction

Motor-evoked potential (MEP) has been the primary response in quantifying the effects of transcranial magnetic stimulation (TMS) since its innovation (Barker et al. 1987; Di Lazzaro et al. 2004; Rothwell 1997). By varying target areas and stimulation protocols, TMS-induced MEP has been utilized in various fields of neuroscience, such as studies of altered neural activities or patients with motor deficits (Säisänen et al. 2015; Vucic and Kiernan 2017).

Targeting TMS pulses to the primary motor cortex (M1) initiates descending waves in the corticospinal tract and evokes MEPs in the contralateral muscles (Devanne et al. 1997; Rothwell 1997; Ziemann 2020). TMS pulses at high stimulation intensities can directly excite neuronal axons, resulting in a D-wave with an onset latency comparable to electrical stimulation. TMS pulses also excite indirect trans-synaptic activations of inter-neurons at the stimulated area, so-called I-waves, which have a ~ 2 ms longer latency than the D-wave (Rossini et al. 2015; Rothwell 1997). In addition, if the nearby cortical regions are also stimulated, such as the premotor cortex, the excitation mediated from these areas to M1 via long-range cortico-cortical fibers results in later I-waves (Dum and Strick 2005; Ziemann 2020).

Furthermore, the direction of the TMS-induced electric field (E-field) strongly affects the recruitment of the D and I-waves and the resulting MEPs (Conforto et al. 2004; de Goede et al. 2018; D’Ostilio et al., 2016; Kallioniemi et al. 2015b; Laakso et al. 2013). D-wave is most likely produced with the lateral-medial current direction, at which I-waves are suppressed. The posterior-anterior (PA) current direction induces all early and late I-waves. In contrast, the anterior-posterior (AP) current direction only produces late I-waves, which result in MEPs with 2–3 ms longer latencies compared to MEPs induced by PA current direction (Derosiere et al. 2020; Di Lazzaro et al. 2001; Hannah and Rothwell 2017). These orientation-selective characteristics of TMS-induced motor responses originate from how TMS activates neurons. The activation happens by axonal excitation when the potential difference between two points along the axon reaches a specific value, which occurs preferentially at axon bends or terminations, suggesting that the preferential site of activation is along the sulcal banks instead of at the gyral crowns (Abdeen and Stuchly 1994; Fox et al. 2004; Ilmoniemi et al. 1999). This model is supported by a combined TMS and positron emission tomography study showing that the M1 sulcal bank exhibited greater cerebral blood flow during TMS, even though the gyral crown was exposed to a stronger E-field (Krieg et al. 2013). However, recent research has cast doubt on this model, suggesting that the preferred site of TMS activation lies in the upper parts of the gyrus, using computational models at the macroscopic (Gomez–Tames et al. 2020) and neuronal levels (Aberra et al. 2020).

MEPs are susceptible to considerable variation as the coil is rotated, and each MEP feature might have a different optimal coil location and orientation (Kallioniemi et al. 2015a; Richter et al. 2013). MEP amplitude and onset latency are the most studied. In contrast, other features, such as MEP polyphasia and duration, are yet to be investigated. Highly polyphasic MEPs have been reported in several movement disorders and motor neuron diseases (Chowdhury et al. 2015; Kohara et al. 1999; van der Salm et al. 2009). However, the behavior of these waveform features in healthy individuals under varying stimulation conditions has not been systematically characterized. Establishing normative patterns of MEP waveform morphology, timing, and size-related features across coil orientations is necessary before these features can be meaningfully interpreted in clinical populations.

The probability of evoking an MEP increases monotonically with increasing stimulus intensity, following a sigmoidal curve, with little change in the MEP waveform (Devanne et al. 1997), and is widely used in defining the threshold for corticomotor excitation, the so-called resting motor threshold (rMT) (Groppa et al. 2012; Kammer et al. 2001; Kimiskidis et al. 2004). The commonly used coil orientation for measuring rMT on M1 is 45° from the parasagittal line. However, it has also been suggested to be perpendicular to the central sulcus, thus considering the individual variability in sulcal geometry (de Goede et al. 2018; Di Lazzaro et al. 2001).

This study aimed to investigate how coil orientation impacts the MEP features relating to its size, timing, duration, and shape in four levels of analysis (intra-session, inter-session, inter-subject, and features grouped from all MEPs). Principal Component Analysis (PCA) was also used to examine the consistency of MEP waveforms across sessions and subjects. In addition, we assessed MEP-induction tendency by counting MEPs at different coil orientations.

## Materials and Methods

### Subjects and Measurements

Data from nine healthy right-handed volunteers (6 females aged 21–29 years) were included. The study was approved by the Research Ethics Committee of the Northern Savo Hospital District (permission 59/2012). The research was conducted in accordance with the principles of the Declaration of Helsinki and local statutory requirements. All participants gave written informed consent to participate in the study.

T1-weighted structural magnetic resonance images using a 3T scanner (Philips Achieva, The Netherlands) were obtained for each subject before the navigated TMS experiment. TMS stimulator (eXimia version 3.2.2, Nexstim Plc, Helsinki, Finland) with a figure-of-eight coil and system-integrated and stimulus-locked six-channel electromyography (EMG) device were used in the experiments.

During the measurements, the subjects sat awake on a comfortable chair, leaning backward with their head resting and in a stabilized position. The coil was manually rotated by a researcher using the navigation software to improve its location, tilt, and orientation, thereby improving accuracy and stability. In addition, the navigation software included a so-called aiming tool that guided the user to move the coil to the target location and orientation that had been previously defined and set in the software while also enabling in-plane rotation of the coil. The surface EMG was recorded from the right first dorsal interosseous (FDI) using Ag-AgCl electrodes in a belly-to-tendon montage. The EMG was sampled at 3 kHz with a resolution of 0.3 µV, and the voltage ranged from − 7.5 mV to + 7.5 mV.

First, the cortical motor representation area of the right FDI was mapped to locate the ‘hotspot,’ where the highest-amplitude MEP was observed in the EMG. During the mapping, the coil was held perpendicular to the nearest sulcus. The coil was rotated at the hotspot to estimate the optimal coil orientation (*R*_*0*_) after stimulation pulses were delivered at various angles. *R*_*0*_ was defined at the angle producing the largest MEP in the right-hand FDI. *R*_*0*_ was individually estimated for each subject and was about 56.7 ± 11.5 ° from the parasagittal line. Afterward, the rMT was determined at the hotspot location using the TMS Motor Threshold Assessment Tool (MTAT 2.0.) (Awiszus and Borckardt 2012).

Three sessions were performed on each subject in a single visit to study the variability of MEP features intra- and inter-session, and inter-subject. Each session contained 120 biphasic stimuli at 120% of the rMT. The first two sessions were conducted consecutively at the same hotspot and combined into a single session (Session 1). Session 3 followed immediately after, with only a few minutes’ rest and the time required to remap the hotspot. The hotspot was remapped to assess short-term inter-session consistency under conditions in which minor differences in hotspot localization are expected. The coil angle was intentionally varied, with approximately a pulse every 2.25 degrees within the rotated sector between − 135 ° and 135° from the optimal coil rotation angle. To assist in changing the coil angle, the rotation was conducted in 45-degree sections, with 20 pulses at approximately even intervals, i.e., 2.25-degree changes. The order of stimulated sectors was randomized. The minimal timing between two pulses was 4.4 s, with a mean and standard deviation of 8.7 ± 3.1 s, to avoid carry-over effects (Julkunen et al. 2012). The location of the maximum E-field at the hotspot-level peeled surface demonstrated only minor variation, which was controlled online via the navigation software. The variation of hotspot location within one session was 2.3–4.2 mm. The distance between the Session 1 and Session 3 hotspots, i.e., the initial and remapped hotspots, ranged from 3.0 to 9.6 mm. The intra-session variation in coil location on the scalp was 2.7–5.1 mm (3.7 ± 0.6 mm) from the hotspot.

### Data Preprocessing

We analyzed the data offline with MATLAB software (version R2016b; MathWorks Inc., Natick, Massachusetts, USA). Coil information, including the coil location, coil direction, and the coil surface normal vector, was extracted from each session’s navigation log files and used to measure the coil angle for each stimulation. It was then assigned to each response, along with the related EMG recording, using timing markers and MEP amplitudes.

The EMG recording was preprocessed using a 250-Hz generalized Butterworth low-pass filter (symmetric filter with the numerator order of 50) (Selesnick and Burrus 1998) and a 50-Hz notch filter. Each response was extracted from 50 ms before the stimulus marker to 250 ms. The smoothness-priors approach removed the baseline drift from individual MEPs (Tarvainen et al. 2002). We excluded MEPs with pre-stimulus activity > 20% of the MEP’s peak-to-peak amplitude and small MEPs with a peak-to-peak amplitude lower than 50 µV.

### MEP Features

MEP features were extracted using an automated MATLAB algorithm (Nguyen et al. 2019). Those included were peak-to-peak amplitude (*Amp*), onset latency (*Lat*), terminal-excluded duration (*eDur*), terminal-included duration (*iDur*), area-under-the-curve (*AUC*), Thickness (*Thickness*), and Size Index (*SizeIndex*). *eDur* is the duration from the MEP onset to the baseline-crossing point of the last prominent MEP peak. *iDur* is the time interval from the MEP onset to the timing point where the MEP curve returns to its baseline, including the low-frequency, low-amplitude wave terminal. *Thickness* and *SizeIndex* are size-related features; *Thickness* is calculated as the ratio of *AUC* and *Amp*, whereas *SizeIndex* is calculated as the sum of *2log*_*10*_*(Amp)* and *Thickness* (Sonoo and Stålberg 1993). Thickness and SizeIndex were originally proposed to characterize motor unit potential waveforms beyond amplitude and duration (Sonoo and Stålberg 1993). They were included here to capture aspects of the MEP waveform beyond peak-to-peak amplitude, as features related to waveform shape may reflect different aspects of corticospinal activation. In addition, the two largest turns’ timings (*T1T*, *T2T*) and amplitudes (*T1A*, *T2A*) were included, along with the timing difference of these turns (*timeDiff*) and the ratio of their amplitudes (*ampRatio*). These features describe four aspects of an MEP: its size (*Amp*,* SizeIndex*,* AUC*,* T1A*,* T2A*), its points of time (*Lat*,* T1T*,* T2T*), its duration (*iDur*,* eDur*,* timeDiff*), and its shape (*Thickness*,* NT*,* NP*,* ampRatio*).

**Fig. 1:**
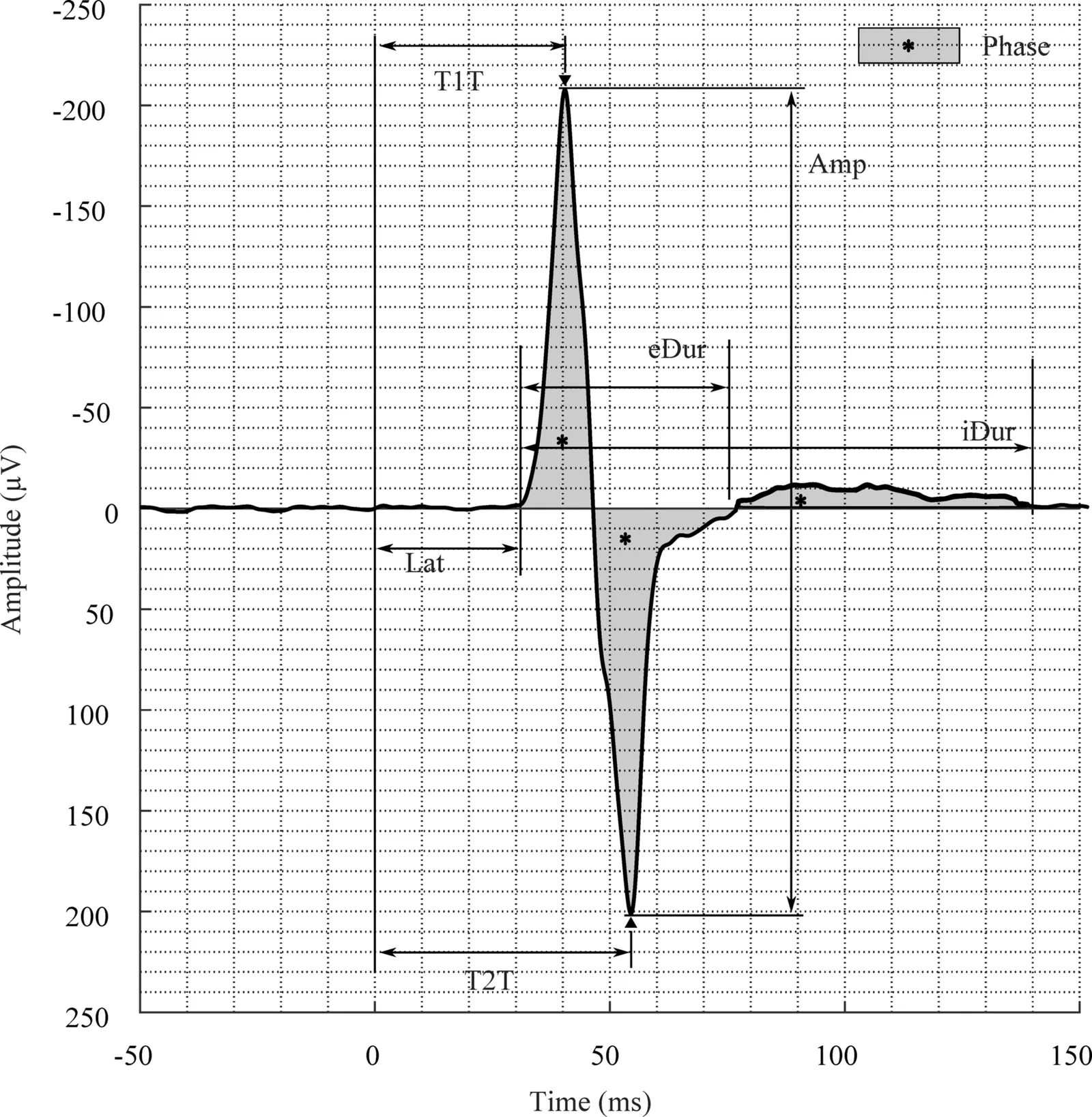
Representative MEP waveform with the extracted parameters indicated. Onset latency (*Lat*) is the interval from the stimulus, i.e., the transcranial magnetic stimulation pulse (0 ms), to the MEP onset. The timings of the two largest turns (*T1T*, *T2T*) are measured from the stimulus to the first and second largest turns; their amplitudes are *T1A* and *T2A*, their timing difference is *timeDiff*, and the ratio of their amplitudes is *ampRatio*. Peak-to-peak amplitude (*Amp*) is the voltage difference between the most positive and most negative peaks. Terminal-excluded duration (*eDur*) runs from the MEP onset to the baseline-crossing of the last prominent peak before the terminal; terminal-included duration (*iDur*) runs from the MEP onset to the final return to baseline, including the low-frequency, low-amplitude terminal. Turns are direction changes of the waveform and phases (shaded areas with asterisks) are the segments between successive baseline crossings; their counts are the number of turns (*NT*) and number of phases (*NP*). Area under the curve (*AUC*) is the area under the absolute MEP within iDur. *Thickness* is the ratio of *AUC* to *Amp*, and *SizeIndex* is calculated as 2·log10(*Amp*) + *Thickness*

The MEP features fall into four categories according to their origin. Peak-to-peak amplitude (*Amp*) and onset latency (*Lat*) are standard, guideline-supported measures in TMS reporting (Groppa et al. 2012; Rossini et al. 2015). Terminal-included duration (*iDur*), area-under-the-curve (*AUC*), and the numbers of turns (*NT*) and phases (*NP*) underlying polyphasia are established in quantitative EMG and motor unit potential analysis and have been reported in clinical MEP studies (Chowdhury et al. 2015; Kohara et al. 1999; Sonoo and Stålberg 1993; van der Salm et al. 2009). Terminal-excluded duration (*eDur*) was introduced in our earlier work (Nguyen et al. 2019), and *Thickness* and *SizeIndex* were adapted from motor unit potential analysis (Sonoo and Stålberg 1993) and likewise applied to MEPs in that work. The timing (*T1T*, *T2T*) and amplitude (*T1A*, *T2A*) of the two largest turns, their timing difference (*timeDiff*), and their amplitude ratio (*ampRatio*) build on the turns concept from quantitative EMG and were defined in this study. The main extracted parameters, including the less standard ones, are indicated on a representative MEP waveform in Fig. 1.

An MEP with more than two turns or more than two phases was considered complex. Because the number of turns (*NT*) and the number of phases (*NP*) are nominal, the percentage of MEPs containing two turns (*f*_*NT = 2*_), having two phases (*f*_*NP = 2*_), and being a complex MEP (*f*_*NT > 2|NP > 2*_) were analyzed; they were calculated by the number of these MEPs divided by the total number of MEPs in each session. The same method was used to compute the proportion of MEPs induced in each 10-degree segment by dividing the number of MEPs evoked in that segment by the total number of MEPs. These proportions were used to compare MEP appearance across different coil orientations.

### Statistical Analysis

The Kolmogorov-Smirnov test for normality was applied to all feature sets. The null hypothesis of normality was rejected in most feature sets based on this test. Hence, the MEP features’ statistics were shown in median and median absolute deviation. Other parameters, such as coil angle, were reported as the mean ± 1 standard deviation. No correction for multiple comparisons was applied; the statistical analyses were exploratory, and the results should be interpreted accordingly.

Four MEP analysis levels were performed. Analysis at the:

- intra-session level evaluated the relation of features among MEPs within a session. The quartile-based Coefficient of Variation (*qCoV*) of MEP features was calculated to assess the dispersion of feature values within each session; it is the ratio of the data’s interquartile range to its median, expressed as a percentage. In addition, Spearman’s correlation coefficient was used to assess correlations between MEP features within each session. Poor, moderate, good, and excellent correlation coefficients were defined as less than 0.50, between 0.50 and 0.75, between 0.75 and 0.90, and greater than 0.90, respectively (Koo and Li 2016).
- inter-session level evaluated the relation of features among sessions of each subject using the two-sided Wilcoxon rank-sum test on the equality of the feature median between two sessions of each subject.
- inter-subject level evaluated the relation of features among subjects. The intraclass correlation coefficient (*ICC*) was used to verify the inter-session repeatability of each feature across subjects. We used the 95% confidence interval, single rating, absolute-agreement, and two-way random-effect models across median values of each feature in two sessions across nine subjects (Koo and Li 2016) using the *irr* package in R (version 3.6.3; R Foundation for Statistical Computing, Vienna, Austria).
- group level investigated the relationship of features grouped from all MEPs. Histogram, feature plots, and Spearman’s correlation coefficient were included in a correlation matrix using the *ggcorr* package in R.

### Gaussian Fitting

The MEPs produced at close coil orientations are expected to be similar in amplitude. Thus, the effect of the coil orientation on *Amp* is gradual. To reduce MEP variability, the impact of coil orientation on *Amp* was studied by fitting a Gaussian curve to all data points collected during a single session, thereby eliminating bias from any single value.

This study used two types of Gaussian fitting: a normal Gaussian function was fitted to *Amp* as a function of stimulation angle, whereas two Gaussian functions were fitted to the MEP-inducing proportion (double-Gaussian fitting). The Levenberg–Marquardt algorithm was used to solve the nonlinear least-squares problem to estimate the Gaussian parameters (Levenberg 1944; Marquardt 1963).

### Principal Component Analysis

PCA was used to assess the consistency of the MEP waveform across coil rotation. To remove *Amp*’s dependency on coil orientation, each MEP was normalized so that its *Amp* was equal to 1. This was done by dividing each MEP sample by the MEP’s *Amp*. Then, PCA was performed on each of these normalized datasets as in (Nguyen et al. 2019).

The normalized dataset $\:\boldsymbol{X}$ consists of * p* MEPs of length * n* samples as columns:

1 $$\boldsymbol{X}=\left[\begin{array}{ccc}{x}_{\mathrm{1,1}} & \cdots & {x}_{1,p}\\ \vdots & \ddots & \vdots \\{x}_{n,1}& \cdots & {x}_{n,p}\end{array}\right]$$

Then, the correlation matrix of $\:\boldsymbol{X}$ was calculated:

2 $$\:\boldsymbol{R}=\frac{1}{p-1}\boldsymbol{X}{\boldsymbol{X}}^{T}$$

Thirdly, the eigenvectors and eigenvalues of $\:\boldsymbol{R}$ were obtained by

3 $$\:\boldsymbol{R}\boldsymbol{\varLambda\:}=\boldsymbol{\varLambda\:}\boldsymbol{V}$$

where $\:\boldsymbol{\varLambda\:}$ contains the eigenvalues in its diagonal, and $\:\boldsymbol{V}$ contains the corresponding eigenvectors as columns. $\:\boldsymbol{V}$ was sorted by eigenvalues in descending order. The *i*^*th*^ column of ***V*** is called the *i*^*th*^ principal component (*PC*) and denoted by *PC*_*i*_. From the eigenvalue, the total variation that *m* PCs account for (*VAF*) was calculated by:

4 $$\:{\varPi\:}_{m}=\sum\:_{i=1}^{m}{\pi\:}_{i}=\frac{1}{tr\left(\boldsymbol{R}\right)}\sum\:_{i=1}^{m}{\lambda\:}_{i}\bullet\:100\%$$

where $\:{\pi\:}_{i}$ is the variance the *PC*_*i*_ accounts for, $\:tr\left(\boldsymbol{R}\right)$ is the trace of the correlation matrix $\:\boldsymbol{R}$, and $\:{\lambda\:}_{i}$ is the *i*^*th*^ eigenvalue (Jolliffe and Cadima 2016).

These *PCs* are linearly independent and can be used as basis vectors to reconstruct the dataset using linear least squares to solve the coefficients for each *PC* (Nguyen et al. 2019). The *VAF* of a *PC* is the proportion of the dataset that is explained by that basis vector. If the *VAF* of a *PC* is high, that PC waveform is commonly shared in the dataset. Afterward, cross-correlation of the first PC from each subject’s two sessions was computed to compare intra- and inter-subject similarity.

## Results

### Feature Variation and Its Relation to Coil Orientation

The size-related features, *Amp*, *AUC*, *SizeIndex*, *T1A*, and *T2A*, correlated strongly at intra-session and group levels (*r > 0.90*,*p < 0.001*). Therefore, we only used *Amp* as the representative of this group. This analysis represented nine features: *Amp*, *Lat*, *eDur*, *iDur*, *T1T*, *T2T*, *timeDiff*, *ampRatio*, and *Thickness*. Two additional features were the percentage of MEPs having two turns *f*_*NT = 2*_, and MEPs having two phases *f*_*NP = 2*_ in each session.

#### Intra-Session Level

**Fig. 2:**
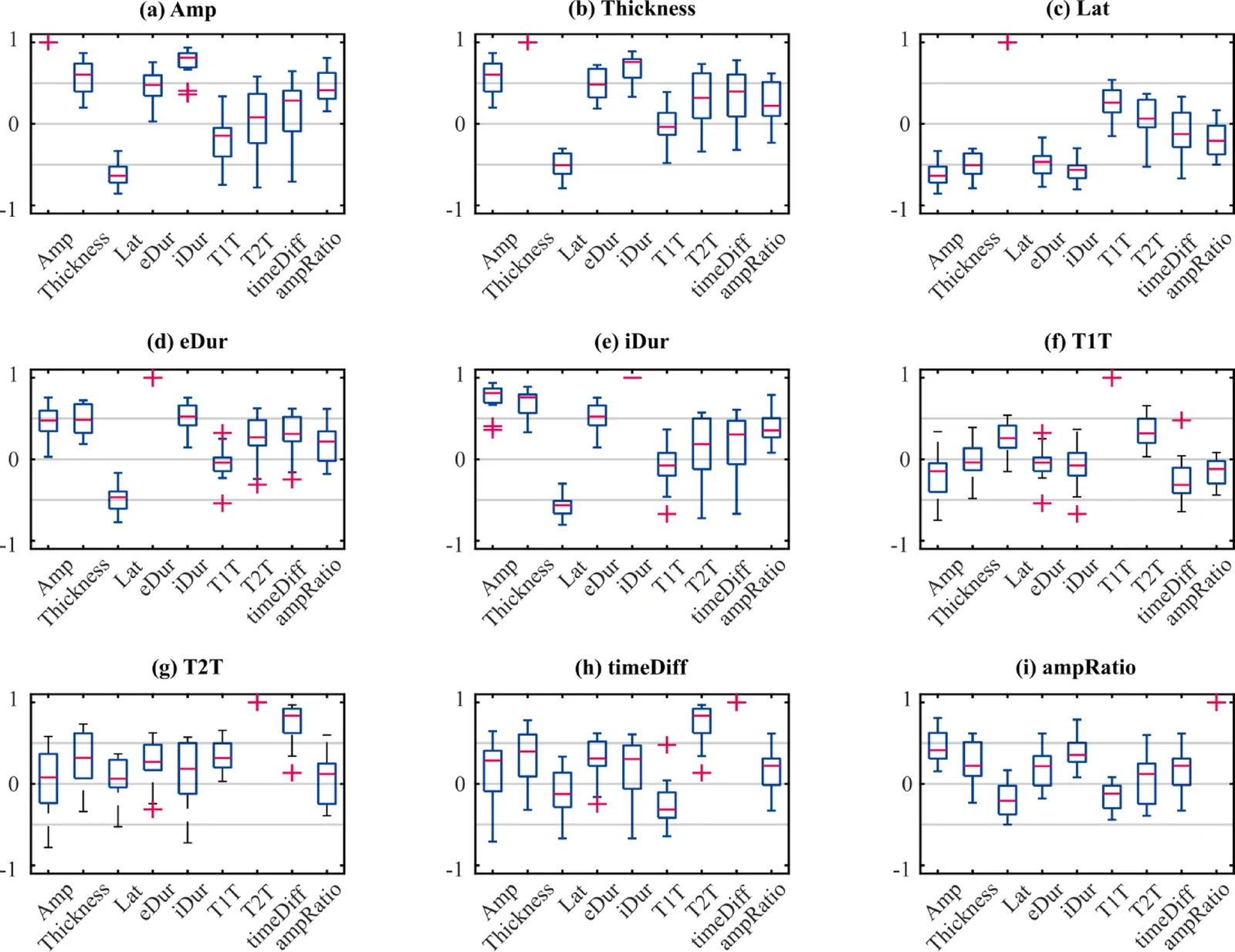
Correlations between MEP features at the session level. Each boxplot summarizes the Spearman correlation coefficients for each feature with the other features, calculated across all 18 datasets. (**a**) represents 18 correlation coefficients of *Amp* with other features; the boxplots of *Thickness*, *Lat*, and *iDur* have medians greater than 0.5, indicating moderate to strong correlations between *Amp* and these features. Similar correlations were also shown in (**b**), (**c**), and (**e**). Also, *T2T* and *timeDiff* had a good correlation (g and h) because *timeDiff* is the timing difference between *T1T* and *T2T*, and *T1T* is mostly constant. Amp: amplitude; Lat: onset latency; eDur: terminal-excluded duration; iDur: terminal-included duration; T1T: timing of the first significant turn; T2T: timing of the second significant turn; timeDiff: timing difference of T1T and T2T; ampRatio: ratio of the two turns

**Fig. 3:**
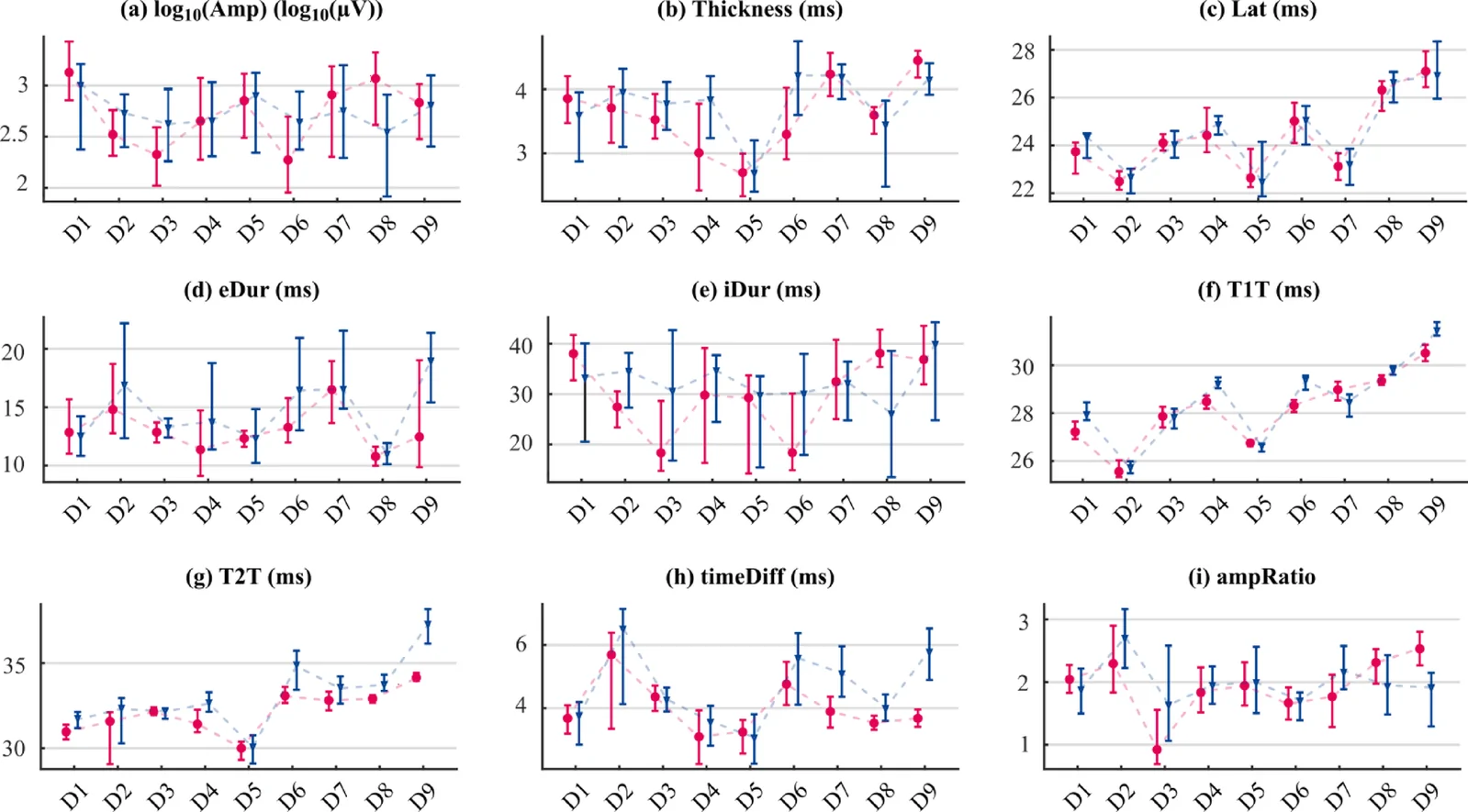
Errorbars of feature values in both sessions of each subject (D1-D9). Session 1 (which consists of the two sessions at the same hotspot) is in pink, Session 3 (the session at the remapped hotspot) is in blue. The plots include the features’ medians (circle and dot markers), 75th percentile (upper end of the error bar), and 25th percentile (lower end of the error bar). Amp: amplitude; Lat: onset latency; eDur: terminal-excluded duration; iDur: terminal-included duration; T1T: timing of the first significant turn; T2T: timing of the second significant turn; timeDiff: timing difference of T1T and T2T; ampRatio: ratio of the two turns

Good between-feature correlations were observed among *Amp*, *Lat*, *iDur*, and *Thickness* (Fig. 2). *T2T* and *timeDiff* also correlated at the session level because *timeDiff* is the timing difference between *T1T* and *T2T*, and *T1T* was mainly consistent. Coil orientation affected MEP features differently (Fig. 3, Appendix A.1). *T1T*, *T2T*, and *Lat* were the least affected, as they had the smallest *qCoV* (Table 1), whereas other features varied widely.

**Table 1:** Intraclass correlation (ICC) and quartile Coefficient of Variation (qCoV) of MEP features

Feature	ICC	*p*-value	qCoV (%)
Lat	0.98***	< 0.001	6.2 ± 1.9
T1T	0.94***	< 0.001	1.9 ± 0.7
T2T	0.85**	0.001	4.4 ± 1.7
NT = 2	0.84**	0.001	
timeDiff	0.77**	0.005	30.8 ± 10.9
ampRatio	0.58*	0.041	45.0 ± 19.0
Thickness	0.57*	0.041	26.5 ± 12.5
eDur	0.46	0.090	28.1 ± 11.2
iDur	0.40	0.125	73.2 ± 38.0
NP = 2	0.24	0.257	
log_10_(Amp)	0.10	0.395	31.0 ± 7.6

Amp: amplitude; Lat: onset latency; eDur: terminal-excluded duration; iDur: terminal-included duration; T1T: timing of the first significant turn; T2T: timing of the second significant turn; timeDiff: timing difference of T1T and T2T; ampRatio: ratio of the two turns; NT: number of turns; NP: number of phases; NT = 2: Proportion of MEPs having two turns; NP = 2: Proportion of MEPs having two phases; log10(Amp): 10-based logarithm of Amplitude. ***p < 0.001, **p < 0.01, *p < 0.05

#### Inter-Session Level

**Fig. 4:**
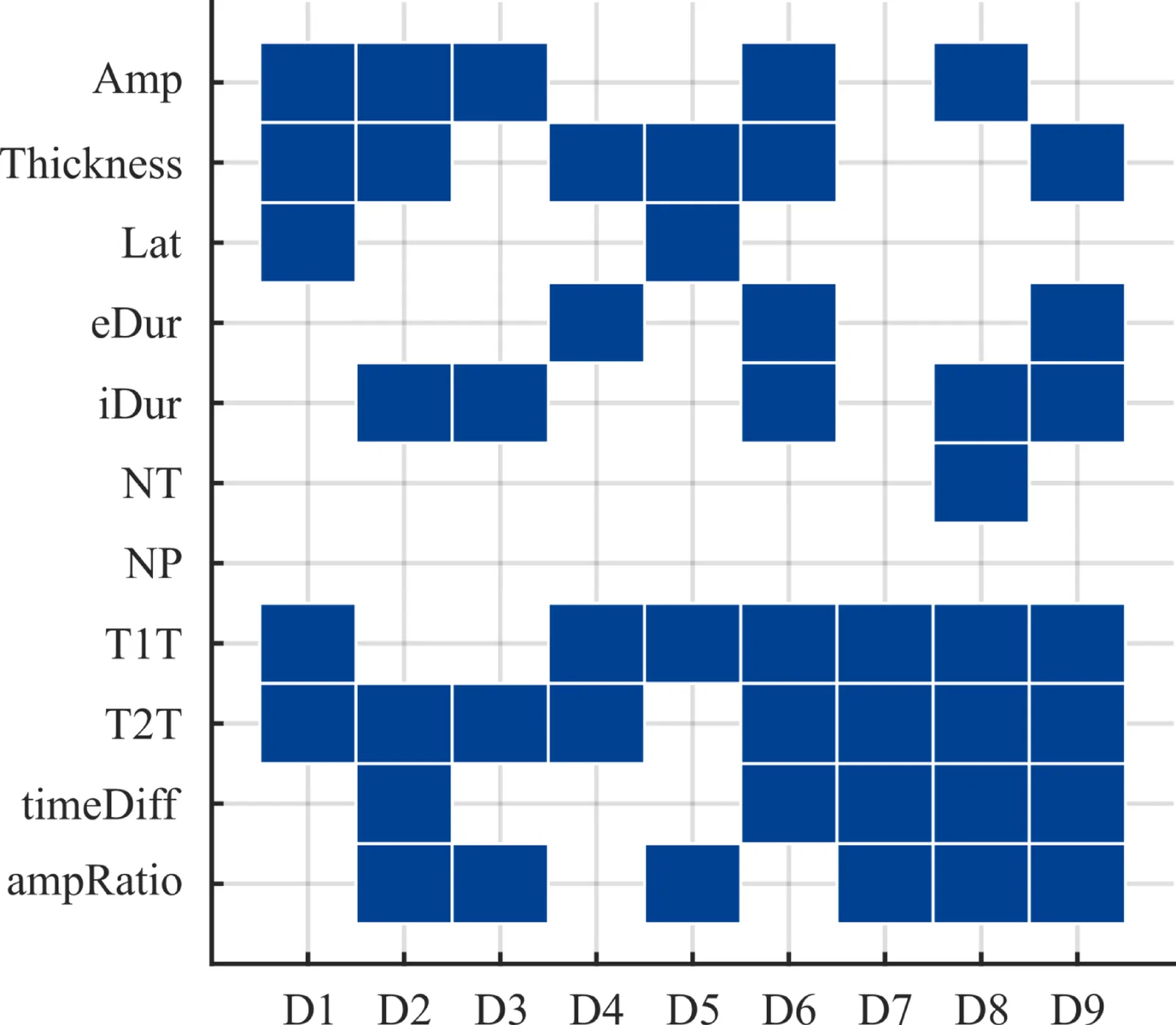
Wilcoxon rank-sum test for the MEP features’ median difference between two sessions (Sessions 1 and 3) of the same subject (D1-D9). Blue boxes represent the rejection of the null hypothesis of equal medians at the 5% significance level. Amp: amplitude; Lat: onset latency; eDur: terminal-excluded duration; iDur: terminal-included duration; T1T: timing of the first significant turn; T2T: timing of the second significant turn; timeDiff: timing difference of T1T and T2T; ampRatio: ratio of the two turns

Figure 4 shows the Wilcoxon rank-sum test of the difference of feature median between two sessions of each subject, where blue boxes represent the rejection of the null hypothesis of equal medians at the 5% significance level. Only *NP* showed equal medians across the two sessions in all subjects, because most MEPs have two phases.

Even though *R*_*0*_ is assumed to be the orientation producing the most prominent peak-to-peak MEP, its inter-session variation was considerable (23.8º ± 15.8º). In contrast, the optimal coil orientations suggested by the normal Gaussian fitting of *Amp* exhibited lower inter-session variation (6.6º ± 4.9º).

#### Inter-Subject Level

**Fig. 5:**
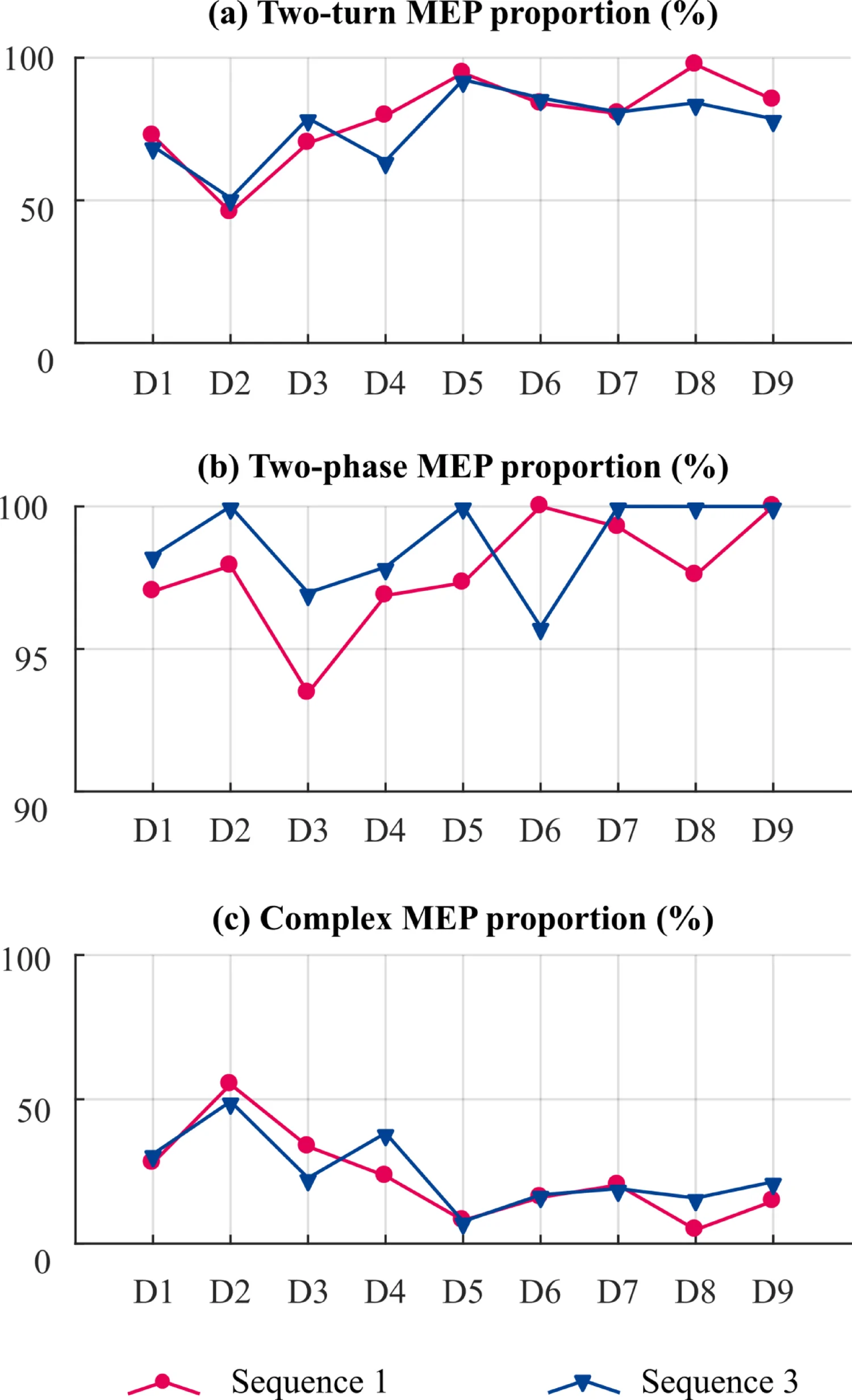
The proportion of MEPs having (a) two turns (NT = 2), (b) two phases (NP = 2), and (c) complex waveform (NT > 2 or NP > 2) in nine subjects. The proportions of MEPs with two turns and complex MEPs are consistent across sessions within a subject (D1-D9).

Table 1 contains the *ICC* of all features, including *f*_*NT = 2*_ and *f*_*NP = 2*_, sorted from the highest to the lowest *ICC*. *Lat*, *T1T*, and *T2T* had excellent *ICC*, whereas *Amp*’s *ICC* was not statistically significant (*p = 0.395*). This is illustrated in Fig. 3, where the variation of feature value in both sessions across all subjects (D1-D9) was plotted. *T1T*, *T2T*, and *Lat* had the lowest inter-session variation and significantly differed across subjects, consistent with the high *ICC* for these features. Furthermore, the *ICC* of *f*_*NT = 2*_ was increased, suggesting the MEP waveform might be subject-specific, while *f*_*NP = 2*_ had a low *ICC* because most MEPs in healthy subjects have two phases (Table 1; Fig. 5).

#### Group Level

The group-level correlation matrix of MEP features is shown in Fig. A.2 (Appendix). It includes plots of features against each other, a histogram of all features, and their correlation coefficients. The features were gathered from all nine subjects with various coil orientations; none of the features were normally distributed at the group level. Upper-right and lower-left triangles show a good linear relationship in four pairs of features: *iDur*-*Amp*, *T1T-T2T*,* Lat*-*T1T*, and *iDur*-*Thickness*. Low correlation was observed between *Amp* and *Lat*, despite their strong intra-session correlation.

### MEP Morphology versus Coil Orientation

The MEP waveforms from all subjects shared common traits despite coil rotation. MEPs with two turns and two phases constituted a large portion of all recorded MEPs. Figure 5 shows that 8/9 subjects have *fNT* = 2 at 81.1 ± 9.3% of the total MEPs; in one subject (D2), one-half of the MEPs had two turns, and the other half had three turns. *f*_*NP = 2*_ were very high, 93.7 ± 3.8%. The proportion of complex MEPs (*fNT* > 2 | *NP* > 2) varied between 8 and 55% across subjects but was intra-subject consistent.

**Fig. 6:**
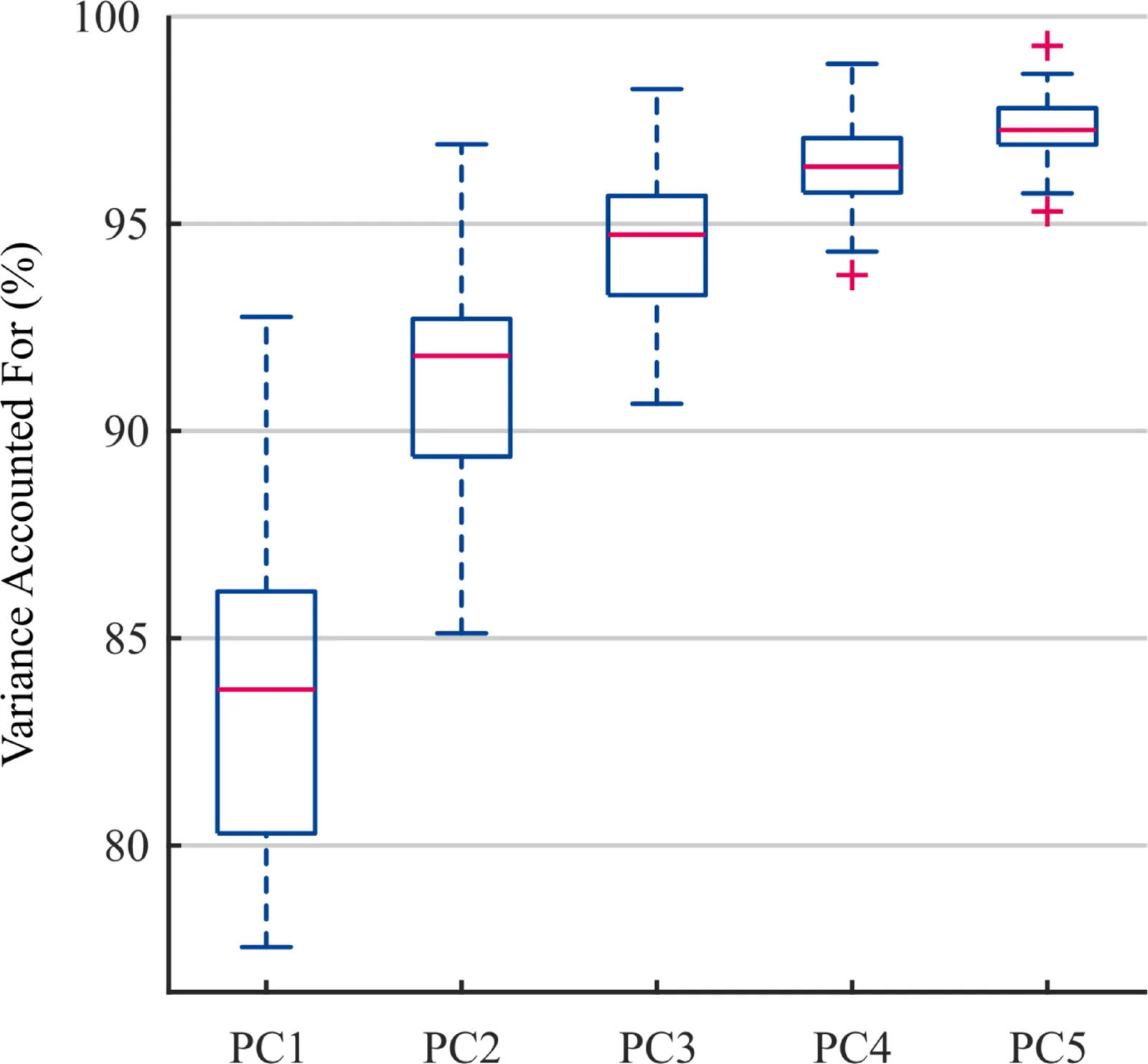
The Variance Accounted For (*VAF*) of Principal components (*PCs*) of normalized datasets. Each boxplot shows the *VAF* for each *PC* across all datasets. The first two *PCs* (*PC1* and *PC2*) account for 91.3% ± 3.0% of the total variation in each dataset, indicating consistency of MEP waveform within each dataset

**Fig. 7:**
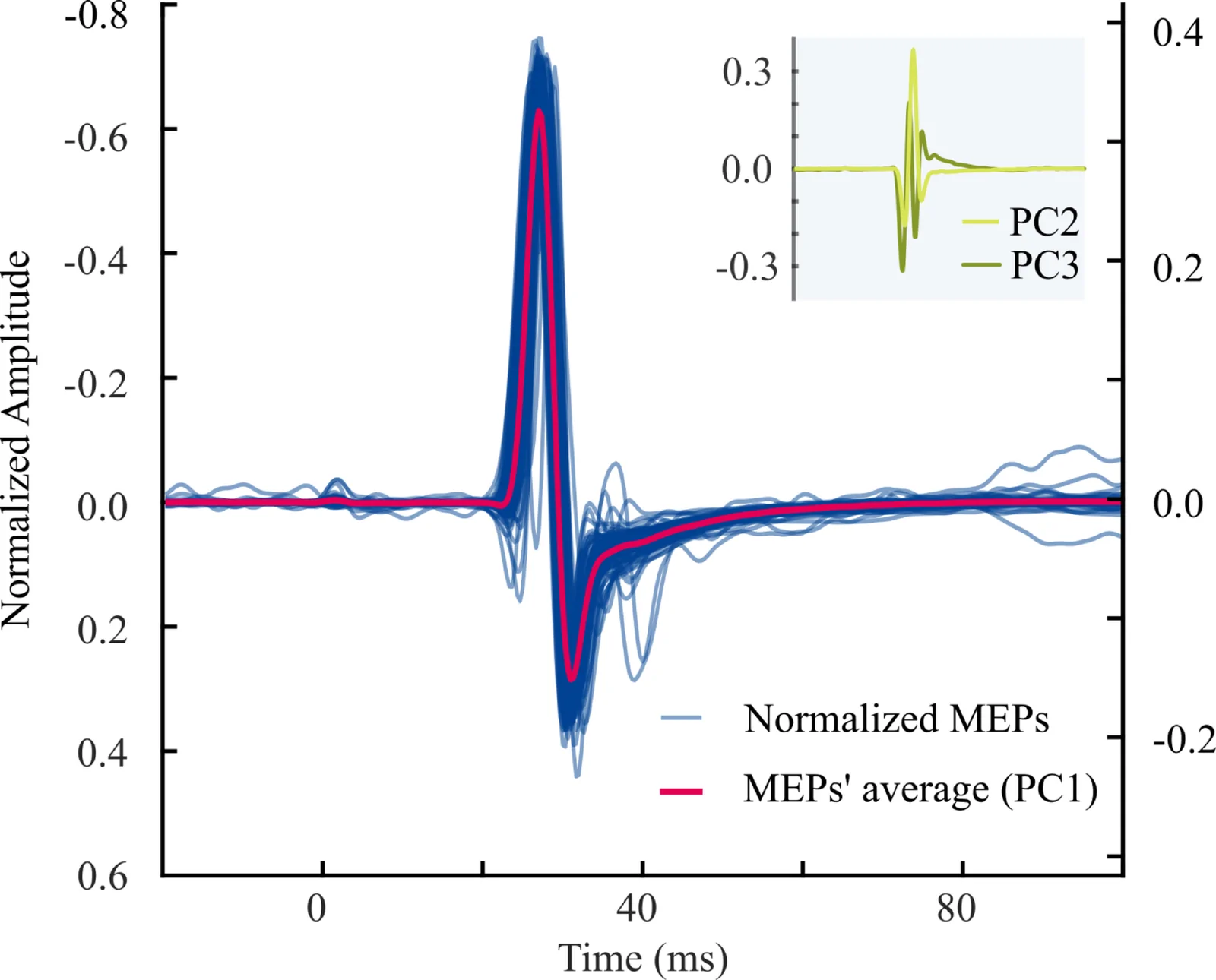
Example of one normalized session. MEPs have a peak-to-peak amplitude of 1 and are plotted in blue lines. The pink line represents the average MEP across that dataset and matches the waveform of the first principal component (*PC1*). The small window in the upper-left corner shows the next two principal components, which capture variations in the MEP waveform. This variation, however, is negligible compared to the *PC1*, as shown in Fig. 6

The PCA output further supported the consistency in MEP morphology within a session. The first two PCs (*PC1* and *PC2*) of normalized MEPs accounted for 91.3 ± 3.0% of the total variation in each dataset (Fig. 6). The MEP waveform remained consistent across sessions and differed slightly across subjects. The inter-session cross-correlation values of *PC1* and *PC2* were 0.98 ± 0.01 and 0.90 ± 0.10, respectively. The cross-correlation values were lower at the inter-subject level, 0.93 ± 0.04 in *PC1* and 0.72 ± 0.19 in *PC2*. An example of a normalized session illustrating this is shown in Fig. 7.

### MEP-Induction Tendency at Different Coil Orientations

**Fig. 8:**
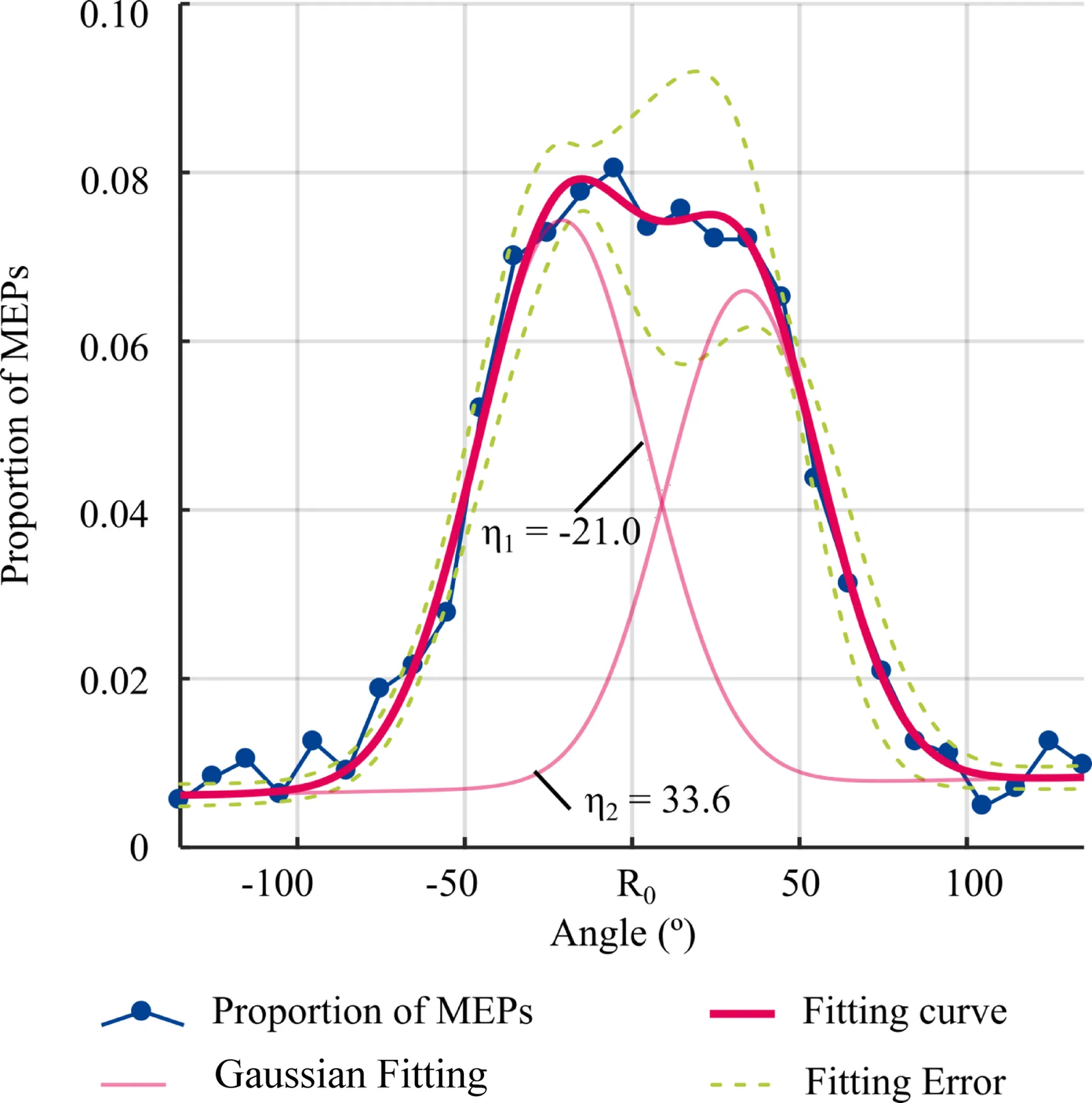
The proportion of MEPs induced at different orientations. Two Gaussian functions (pink) were fitted to the MEP-inducing proportion curve (blue). The expected values of the two Gaussian curves (*η1* and *η2*) indicate that the most MEP-producible orientations were − 21.0 and 33.6º from the optimal coil orientation (*R*_*0*_)

Figure 8 shows the proportion of MEPs induced at each 10º segment, measured in all subjects. The double-Gaussian fitting suggested that MEPs were most likely to be elicited within a -21.0º to 33.6º window with respect to *R*_*0*_, in which *R*_*0*_ was 56.7º ± 11.5º from the parasagittal line. Yet, this pattern was inconsistent at the subject level: 3/9 subjects had the highest number of MEPs induced at 10.0º ± 9.3° from *R*_*0*_, whereas 6/9 subjects had MEPs more frequently at 26.0º ± 11.1° from *R*_*0*_, in either one or both sessions.

## Discussion

This research explored how coil orientation affects the characteristics of MEPs, including magnitude, timing, duration, and complexity. Previous studies have shown that the coil orientation greatly influences the induction of an MEP (Dum and Strick 2005; Kallioniemi et al. 2015a; Rothwell 1997; Ziemann 2020). Yet these results were mainly based on *Amp*. To complement previous findings, we investigated a comprehensive set of MEP features characterizing its size, timing, duration, and shape. We found that while coil orientation strongly affects MEP size, the dominant MEP waveform morphology remains consistent inter- and intra-session and is comparable across healthy subjects.

*R*_*0*_ is the coil angle producing the MEP with the largest *Amp* and shortest *Lat* in TMS practices. Based on epidural recordings, Day et al. (1987) proposed that at *R*_*0*_, the E-field may preferentially activate pyramidal axons directly, producing D-waves, while at oblique angles, transsynaptic activation produces I-waves with 1‑2 ms longer latencies (Day et al. 1987; Rothwell 1997). Although the present study used surface EMG recordings, which cannot distinguish between D-wave and I-wave contributions, *Amp* and *Lat* correlated inversely and moderately within a session, consistent with this theoretical framework (Rothwell 1997). Moreover, *Amp*-coil orientation curves followed normal Gaussian fitting (Kallioniemi et al. 2015a). A coil orientation of 45º with the parasagittal line for *R*_*0*_ was recommended so the E-field would be perpendicular to the central sulcus (Rossini et al. 2015). Still, the optimization of *R*_*0*_ can also be done based on the subject’s gyral anatomy with the aid of neuronavigation (Gomez-Tames et al. 2018; Julkunen et al. 2009). The intra-session *Lat* difference was 1.35 ± 0.42 ms. However, because stimulation was delivered at a constant 120% of rMT determined at the optimal angle, rotating the coil also reduces the effective stimulation intensity at the cortical target. This could contribute to both the decrease in *Amp* and the increase in *Lat*, as higher effective intensities can recruit D-waves that shorten onset latency (Day et al. 1987). The observed covariation of *Amp* and *Lat* across orientations, therefore, cannot be attributed solely to the activation of distinct neural populations. Other features were also affected by the coil orientation to some extent (Fig. 3). The Gaussian fit to the *Amp*-versus-coil-orientation relationship often exhibited a bimodal rather than a unimodal shape, suggesting that different neuronal populations were activated (Kallioniemi et al. 2015a). This bimodal shape could result from multiple possible loci of the largest *Amp* (Kallioniemi et al. 2015a; Reijonen et al. 2020) or due to less cortical volume being exposed to the electrical field as the coil is perpendicular to M1 than being oblique. In certain sessions of this study, a bimodal shape was observed, but its consistency varied among sessions for individual subjects. The MEP occurrence at *R*_*0*_ was lower compared to -30º and 30º coil orientations, so there could be a chance that the most prominent MEP was missed, resulting in a flatter Gaussian curve or even a bimodal curve. However, this effect is unlikely to account for the overall findings.

In particular, *T1T* was the most consistent MEP feature, as it was least affected by coil orientation, with a low *qCoV* and a high *ICC*. This indicates that TMS-evoked corticospinal waves are modulated such that the most prominent MEP peak appears simultaneously regardless of coil orientation. This also describes the low correlation of *T1T* with any other features at the intra-session level (Fig. 2). Yet it showed a moderate correlation with *Lat* and *T2T* at the group level (Appendix, Fig. A.2). At the same time, *Lat* and *Amp* became less correlated, indicating that *T1T* has a different behavior than *Amp* and *Lat* and provides additional information on corticospinal pathway states. Along with *T1T*, other timing-related features, such as *Lat*, *T2T*, and *timeDiff*, also had high ICC. However, the Wilcoxon rank-sum test for median equality showed low inter-session reliability for most MEP features, except NP, which is typically 2 in normal subjects.

The discharge probability of single motor units correlated with the stimulus intensity at a certain angle (Devanne et al. 1997). This discharge probability differs from the MEP-induction tendency at each orientation range of the coil in our study because different neurons are activated at different coil orientations (Kallioniemi et al. 2015a). We rotated the coil while delivering stimuli at a constant and sufficiently high intensity and counted the number of MEPs induced at each orientation segment. Our results showed that the highest number of MEPs was obtained at ~ 30º from *R*_*0*,_ and the MEP-induction tendency at each orientation was uncorrelated with the value of *Amp.*

**Fig. 9:**
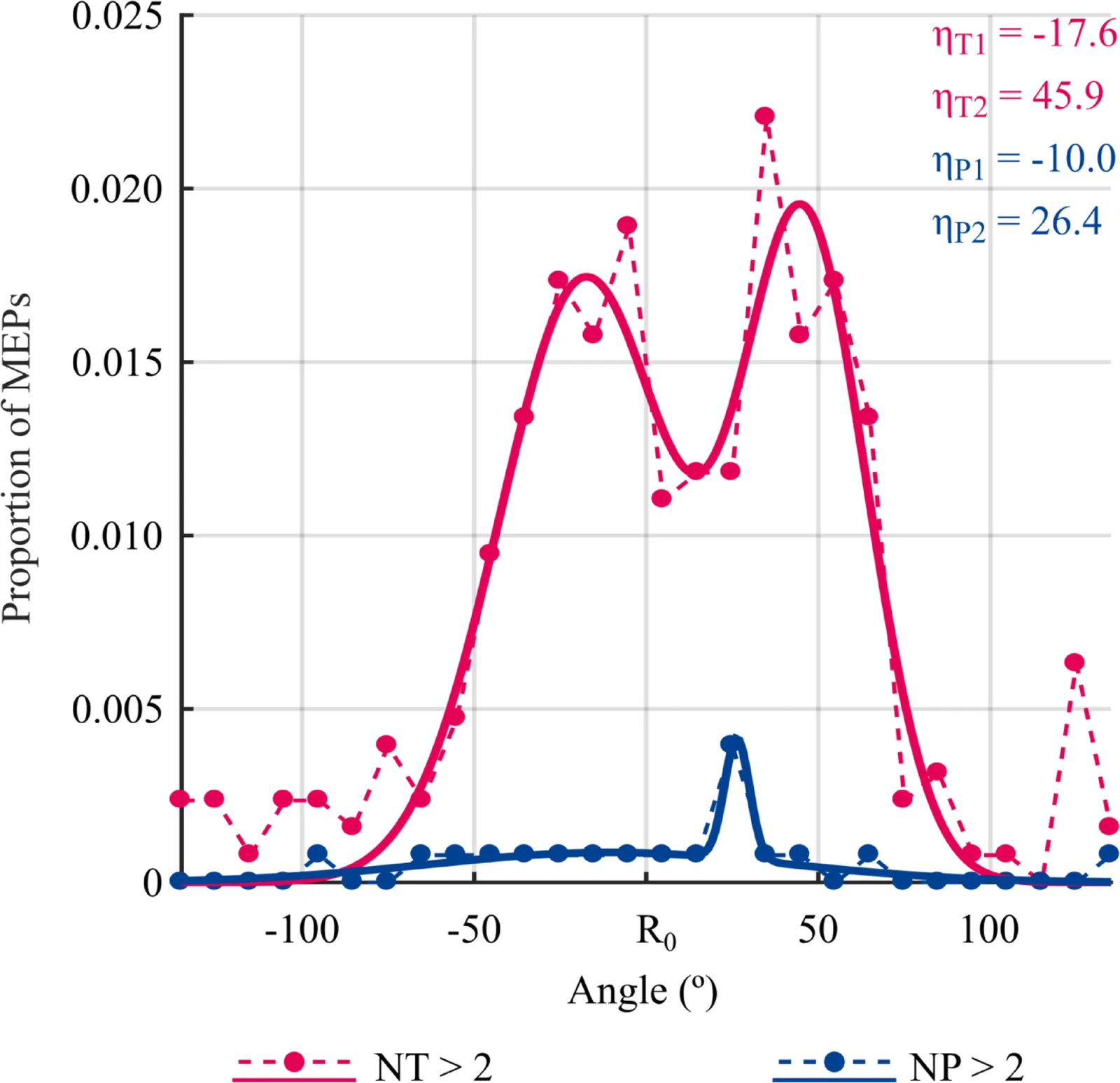
Proportion of complex MEPs having more than two turns (*NT* > 2) (dashed pink line), and two phases (*NP* > 2) (dashed blue line). Two Gaussian functions (pink and blue lines) were fitted to both proportion curves. The expected values of the two Gaussian curves fitted to two-turn MEPs proportion (*ηT1* and *ηT2*) indicate that the complex MEPs having more than two turns are more likely occur at -17.6º and 45.9º from the optimal coil orientation (*R*_*0*_). Complex MEPs with more than two phase peaks peaked between − 10.0 º and 26.4º from *R*_*0*_, however, this proportion was low due to the small number of these MEPs

In addition, the proportion of normal to complex MEPs persists across sessions within a subject but differs across subjects (Fig. 5), and the complex MEPs frequently occurred about 30º from *R*_*0*_ (Fig. 9). This might be due to the significant contribution of the white matter to MEP at the stimulated area (Fox et al. 2004; Rothwell 1997), with contributions from the neighboring grey matter. As the coil rotates away from *R*_*0*_, the misalignment of the E-field and the anisotropy of the white matter at the target increase, which could reduce the effectiveness of direct axonal activation. At the same time, the E-field may cover more grey matter along the sulcus at oblique angles, potentially activating additional cortical regions and contributing to more complex MEP waveforms. However, as the present study relied on surface EMG, these mechanistic interpretations remain speculative and would require epidural recordings to confirm.

We found that the MEP morphology is highly consistent. Our earlier study showed that 95.6% of MEP information was retained within four trials, i.e., within 10 degrees, while 80% was retained within five trials (Nguyen et al. 2019). In the current study, PCA results indicate that the dominant MEP morphology is highly consistent across all levels of analysis, whereas the proportion of complex MEPs depended on coil orientation (Fig. 9). By containing more than 90% of the total variation in each session, the first two PCs are sufficient to form a reliable basis for describing the intrinsic morphology of MEPs in each session. Furthermore, their cross-correlation values at the inter-session and inter-subject levels were very high, indicating consistency in the MEP waveform in healthy subjects. Moreover, slight differences in MEP waveform across subjects can be visually observed and described by the lower inter-subject cross-correlation value of *PC2*.

### Study Limitations

Different coil positions and tilting induce different E-field distributions in the brain (Reijonen et al. 2020). In our study, this effect was minimized by using the neuronavigation software, particularly the aiming tool, to maintain the coil location and its tilt virtually unchanged. The navigation software continuously tracked the coil positioning with respect to the subject’s head and illustrated the real-time E-field changes (Julkunen et al. 2009). In the present study, the E-field calculation was not used for any purpose other than targeting TMS.

In addition, coils with different designs produce the E-field differently in terms of focality, depth of penetration, and distribution (Bottauscio et al. 2016; Deng et al. 2013). Therefore, the findings of our study are limited to figure-of-8 coils, the most common coils used in navigated TMS. However, it is expected that somewhat similar results would be found with other coil types with some added variation. Similarly, the stimulation pulses were delivered in a biphasic waveform, which includes a small backward E-field whose effect was minor compared to the primary forward stimulation. Different results might be obtained with monophasic waveforms. All three sessions were performed within a single visit, and the third session immediately followed hotspot remapping. The present findings therefore reflect short-term, within-visit consistency rather than longer-term test-retest reliability across separate sessions or days, which remains to be established.

Finally, the cohort comprised nine young, predominantly female, right-handed healthy volunteers. While the within-subject design provides many MEPs per participant and supports intra- and inter-session analyses, the small and demographically narrow sample limits generalizability, particularly to clinical populations, for which these features warrant separate validation.

### Conclusions

Overall, this study comprehensively analyzed the effects of TMS coil orientation on MEP features and waveforms. Timing-related features were more robust to coil orientation, whereas size-related features varied considerably as the coil rotated. This may partly reflect the greater robustness of timing features, though rotating the coil inherently changes the effective stimulation intensity at the cortical target, which likely accounts for some of the variation in size-related features. Nevertheless, the occurrence of complex MEPs most frequently at approximately 30° from the optimal orientation (Fig. 9) and the subject-specific consistency of waveform morphology across sessions (Fig. 5) suggest that coil orientation influences MEP waveform properties beyond what would be expected from a simple reduction in effective stimulation intensity. Notably, the highest number of MEPs appeared at ~ 30º from *R*_*0*_, not at *R*_*0*_, where the most prominent MEP is produced. Moreover, most recorded MEPs exhibited highly similar waveforms across sessions and subjects, indicating high within-visit consistency in healthy individuals. Together, these findings highlight the value of examining a comprehensive set of MEP features and waveform morphology beyond peak-to-peak amplitude when characterizing the effect of coil orientation on cortical motor responses.

## Figures and Tables

**Fig. 10:**
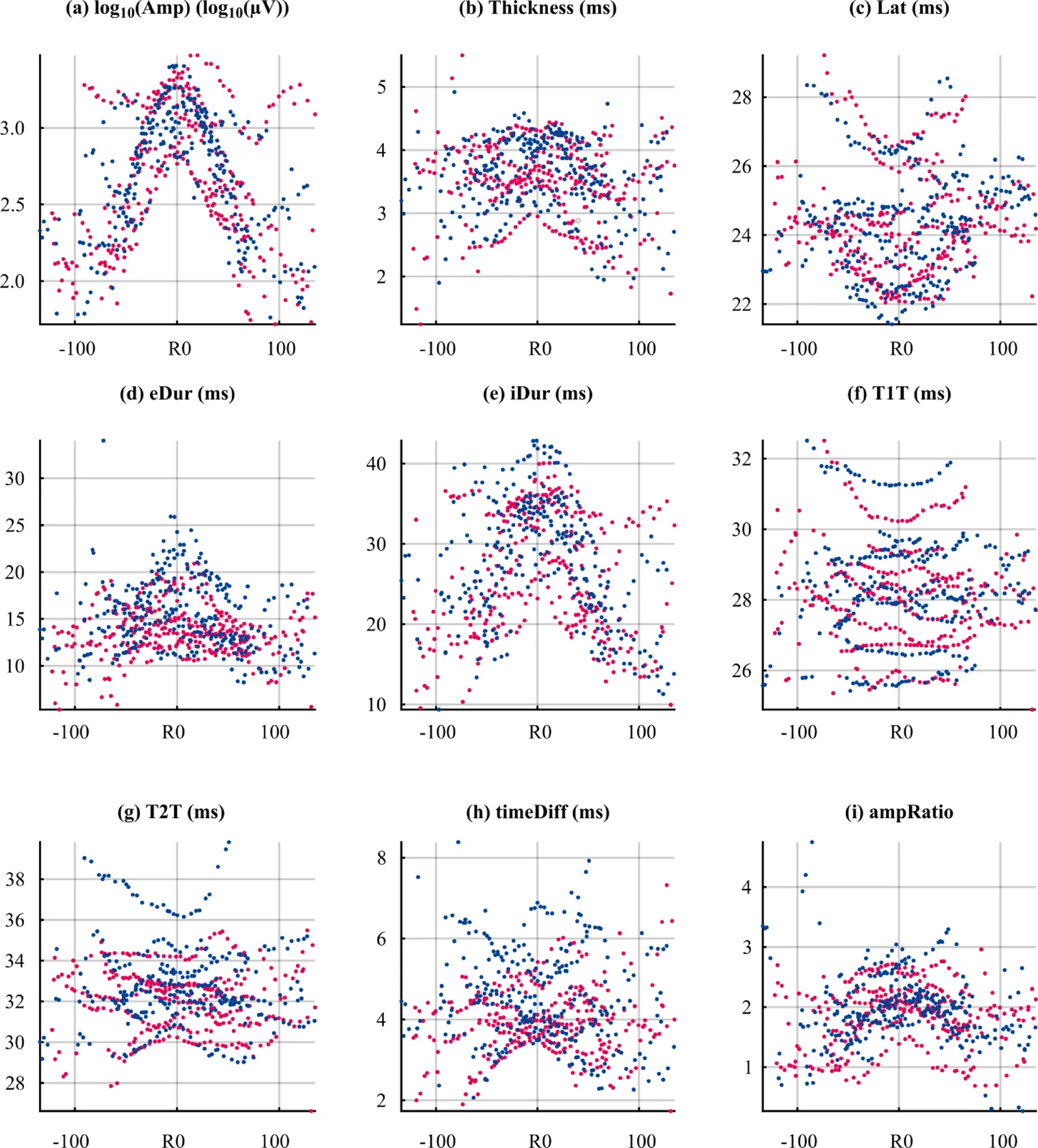
Plots of the average MEP features against the coil orientation; Session 1 (which consists of the two sessions at the same hotspot) is in pink, Session 3 (the session at the remapped hotspot) is in blue. Amp: amplitude; Lat: onset latency; eDur: terminal-excluded duration; iDur: terminal-included duration; T1T: timing of the first significant turn; T2T: timing of the second significant turn; timeDiff: timing difference of T1T and T2T; ampRatio: ratio of the two turns

**Fig. 11:**
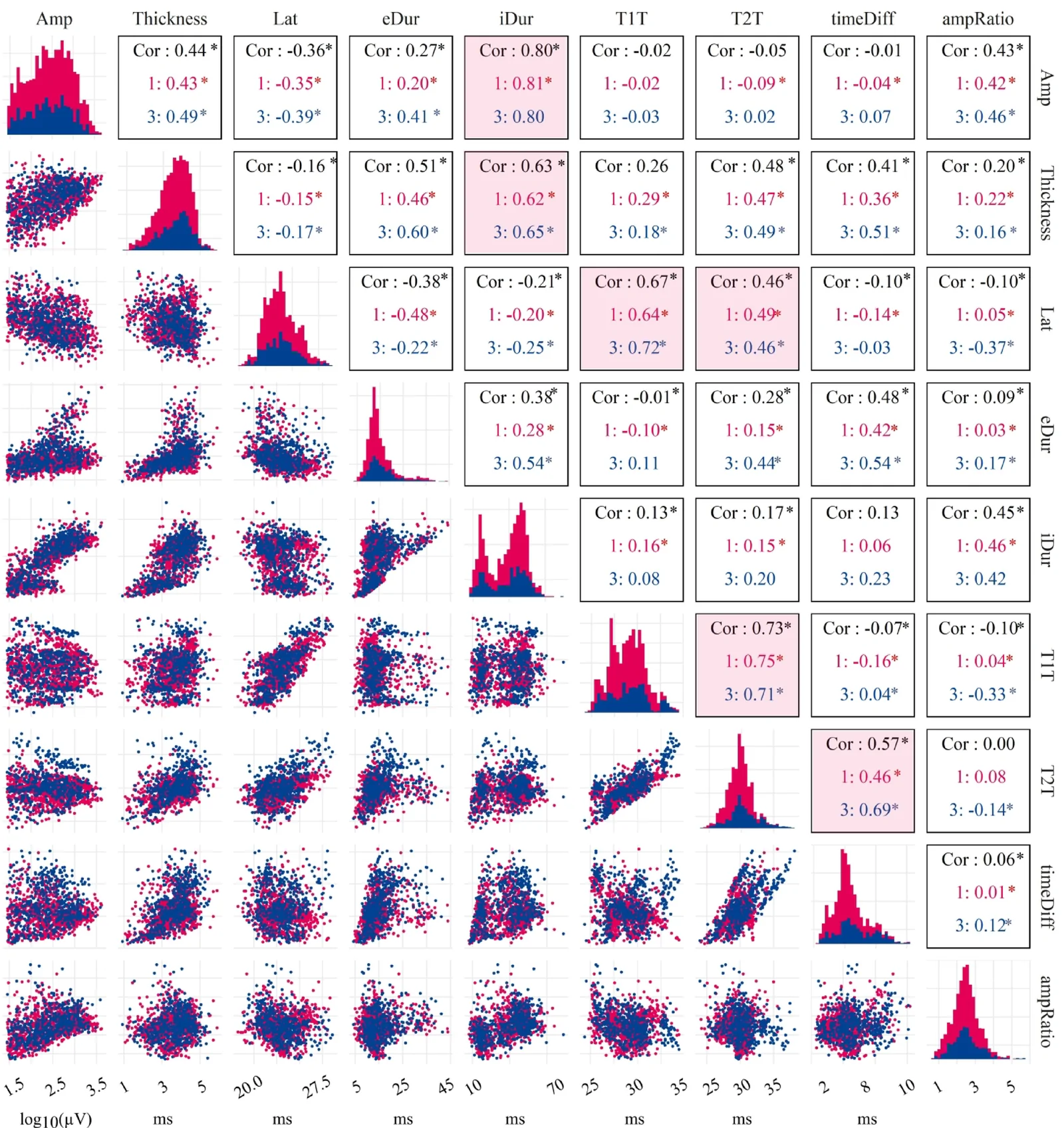
Feature histograms and inter-feature relationships of the correlation coefficients at the group level; Session 1 (which consists of the two sessions at the same hotspot) is in pink, Session 3 (the session at the remapped hotspot) is in blue. The upper triangle contains the correlation coefficients among features: all sessions (black), only the first session (Session 1, pink), and only the third session (Session 3, blue). Pink boxes indicate good to moderate correlations, and asterisks indicate a *p*-value less than 0.05. Feature histograms are plotted on the diagonal, and the lower triangle shows features plotted against each other. Amp: amplitude; Lat: onset latency; eDur: terminal-excluded duration; iDur: terminal-included duration; T1T: timing of the first significant turn; T2T: timing of the second significant turn; timeDiff: timing difference of T1T and T2T; ampRatio: ratio of the two turns

## Data Availability

Data will be made available on request.

## Appendices

See Fig(10, 11)
